# ^1^H NMR Metabolic Profile to Discriminate Pasture Based Alpine Asiago PDO Cheeses

**DOI:** 10.3390/ani9100722

**Published:** 2019-09-25

**Authors:** Severino Segato, Augusta Caligiani, Barbara Contiero, Gianni Galaverna, Vittoria Bisutti, Giulio Cozzi

**Affiliations:** 1 Department of Animal Medicine, Production and Health, University of Padova, 35020 Legnaro (PD), Italy; barbara.contiero@unipd.it (B.C.); vittoria.bisutti@unipd.it (V.B.); giulio.cozzi@unipd.it (G.C.); 2 Department of Food and Drug, University of Parma, 43124 Parma, Italy; augusta.caligiani@unipr.it (A.C.); gianni.galaverna@unipr.it (G.G.)

**Keywords:** alpine pasture, Asiago cheese, ripening time, NMR, canonical discriminant analysis

## Abstract

**Simple Summary:**

Nowadays, alpine cheese from grazing dairy herds has a premium market value because consumers perceive its higher degree of healthiness and sustainability. The authenticity of pasture-based cheese should be safeguarded from local hay-based milk analogues. The study aimed at assessing the reliability of proton nuclear magnetic resonance (^1^H NMR) to discriminate pasture-based alpine Asiago PDO cheeses of different ripening time from similar hay-based samples processed in the same dairy plant. Cheeses were produced from raw milk collected from grazing or hay-fed alpine dairy herds and they were ripened for 2 (*Pressato*), 4 (*Allevo*_4), and 6 (*Allevo*_6) months. Samples of the cheeses were submitted to wet chemistry and nuclear magnetic resonance analysis. The outcomes of the ^1^H NMR spectroscopy were used in a multivariate discriminant procedure. Choline, 2,3-butanediol, lysine, and tyrosine and some residual sugar-like compounds were water-soluble biomarkers of cows’ feeding system. However, the application of ^1^H NMR based metabolomics was an effective fingerprinting method to correctly identify only cheese samples with the shortest ripening period. The classification of more aged cheese samples according to the cows’ feeding system was less accurate likely due to the chemical and biochemical changes induced by a prolonged maturation process.

**Abstract:**

The study was carried out in an alpine area of North-Eastern Italy to assess the reliability of proton nuclear magnetic resonance ^1^H NMR to fingerprint and discriminate Asiago PDO cheeses processed in the same dairy plant from upland pasture-based milk or from upland hay-based milk. Six experimental types of Asiago cheese were made from raw milk considering 2 cows’ feeding systems (pasture- vs. hay-based milk) and 3 ripening times (2 months, *Pressato* vs. 4 months, *Allevo_4* vs. 6 months, *Allevo_6*). Samples (*n* = 55) were submitted to chemical analysis and to ^1^H NMR coupled with multivariate canonical discriminant analysis. Choline, 2,3-butanediol, lysine, tyrosine, and some signals of sugar-like compounds were suggested as the main water-soluble metabolites useful to discriminate cheese according to cows’ feeding system. A wider pool of polar biomarkers explained the variation due to ripening time. The validation procedure based on a predictive set suggested that ^1^H NMR based metabolomics was an effective fingerprinting tool to identify pasture-based cheese samples with the shortest ripening period (*Pressato*). The classification to the actual feeding system of more aged cheese samples was less accurate likely due to their chemical and biochemical changes induced by a prolonged maturation process.

## 1. Introduction

Cheese authentication is a hot topic of interest for producers, consumers and policy makers, with a specific focus to control the labelling claims on high-quality dairy products. Indeed, these products and the Protected Designation of Origin (PDO) ones in particular have distinctive characteristics according to their geographical production area, cows management and feeding system as well as to cheese making procedure [1,2,3].

Asiago PDO cheese is protected “from farm to fork” by an EU regulation (Production Specification PDO ‘Asiago’ [4]), that sets the geographical area of milk origin and the specific guidelines for cheese production. With specific regard to the curd manipulation and ripening time, Asiago cheese can be produced as pressed type, so-called *Pressato*, with a short ripening period ranging from 20 up to 90 days. The *Allevo* type is characterized by a longer ripening time, a harder texture, and a stronger and composite flavor. However, in order to meet the request of young consumers for less strong-flavored cheese, some mountain dairy plants have limited the ripening time of Asiago *Allevo* especially when processing raw milk from grazing dairy herds. Within the basket of Asiago PDO, cheese from alpine grazing herds have been recognized for a lipid and water-soluble fraction rich of functional nutrients and aromatic substances [1,5]. These products have a premium market value because consumers perceive their higher degree of healthiness and sustainability [6,7].

In this scenario, there is an increasing request by both dairy farmers and consumers to safeguard the authenticity of pasture-based cheese from local analogues obtained by milk from cows fed conserved forage. This target requires the development of robust and comprehensively analytical techniques that should support self-inspections by producers or audits by independent certification bodies. Among chemometric techniques, high-resolution proton nuclear magnetic resonance (^1^H NMR) spectroscopy represents a promising tool for metabolic fingerprinting of milk and derivatives as it is able to offer in a single experiment an overview of a wide range of metabolites or metabolite species [8]. This optical-method was applied to commercial samples of cow’s milk in order to discriminate their nutritional properties [9]; it was used to discriminate milk according to cows’ feeding systems [10] or productive method (organic vs. conventional) [11]. Moreover, in the dairy sector it has been used to discriminate the geographical origin of buffalo [12] and cow’s [13] cheese as well as to monitor the ripening process of two of the main Italian PDO cheeses: Parmigiano Reggiano [13] and Grana Padano [14]. The aim of the present study was to assess the reliability of ^1^H NMR technique coupled with multivariate canonical discriminant analysis (CDA) to discriminate pasture-based alpine Asiago cheese of different maturation time from analogues processed in the same dairy plant from hay-based milk. 

## 2. Materials and Methods 

### 2.1. Experimental Design and Cheese-sampling Procedure

The study was carried out in the upland of the so-called Asiago plateau (around 1000 m above sea level) located in the Veneto Region (North-Eastern Italy). In this Alpine area, eight dairy farms with similar herds in relation to cows’ breed, milk yield, and management were selected. Four farms used a pasture-based production system with the summer grazing of cows on a natural pasture (pasture-based treatment) supplemented with a daily amount of around 4.5 kg of dry matter (DM) of concentrate (mixture of maize and barley meal, soybean meal and mineral-vitamin mix). In the other four farms (hay-based treatment), cows were kept indoor and fed total mixed rations based on (dry matter basis) around 45–50% of grass hay from local upland permanent meadows, 5–8% of alfalfa hay and a 42–50% of concentrate (maize and barley meal, soybean and sunflower meal, mineral–vitamin mix). 

Three bulk of milk samples were collected for both pasture-based and hay-based farms group in the middle of July, in the middle of August and in early September. At each sampling time, the bulk milk samples of the two cows’ feeding systems were transferred to the same mountain dairy plant for cheese making. Whole raw bulk milk of the two cows’ feeding systems was separately processed according to the PDO specifications [15] to produce wheels of Asiago: i. *Pressato* (2-mo of ripening); ii. *Allevo_4* (4-mo of ripening), and iii. *Allevo_6* (6-mo of ripening). The ripening process of all samples was carried out in the same storage bay at 10 ± 2 °C and 80–85% relative humidity.

Cheese samples (about 2 kg each) of the 6 types of Asiago obtained according to the 2 cows’ feeding systems (FS) per 3 ripening times (RT) combination were cut as a quarter of a whole block and taken to the laboratory under refrigeration (3 ± 1 °C). Each sample portion was cut across the whole block in order to take into account concentration gradients, avoiding the first 2 cm starting from the rind. 

### 2.2. Chemical and ^1^H NMR Analysis

As previously reported [5], the cheese proximate composition was carried out in triplicate by using the standard International Dairy Federation Methods for moisture, crude protein, water-soluble nitrogen (water-soluble N), crude ash and sodium chloride (NaCl), meanwhile crude fat was analyzed by an accelerated solvent extraction method. The pH was determined twice with a portable pH-meter (KnickPortamess® 911, Berlin, Germany) equipped with a conic electrode (5 mm Ø conic tip, Crison 5232, Modena, Italy) on a homogenized aqueous solution. 

**Figure 1:**
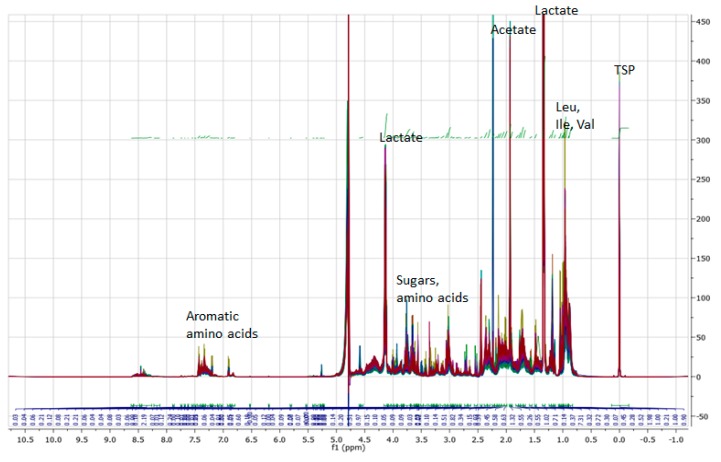
Overlay of ^1^H NMR spectra (600 MHz) of the alpine Asiago Protected Designation of Origin (PDO) samples (D_2_O extracts).

With regard to ^1^H NMR analysis, around 200 mg of each cheese samples were ground and mixed with 1 mL of D_2_O. The mixture was blended with a magnetic blender for 1 h at room temperature. To ensure a complete removal of the apolar component, 100 µL of CDCl_3_ was added. After centrifugation, 600 µL of the supernatant was taken for the analysis. An amount of 100 µL of *3*-(trimethylsilyl)-propionate-d4 (Sigma-Aldrich, Milano, Italy) was used as internal standard (20 mg/10 mL of D_2_O). ^1^H NMR spectra were recorded on a VARIAN INOVA-600 MHz spectrometer (Varian, Palo Alto, CA, USA), equipped with a 5-mm triple resonance inverse probe. Spectra were acquired at 298 K, with 32 K complex points, using a 45° pulse length and 3 s of relaxation delay (d1). 128 scans were acquired with a spectral width of 9595.8 Hz and an acquisition time of 1.707 s. Identification of alpine Asiago PDO ^1^H NMR markers was achieved through 1D and 2D spectra; TOCSY spectra were acquired at 298 K, with 2048 data points. A total of 32 scans was acquired for each of 256 increments, with an acquisition time of 0.155 s. Acquired ^1^H NMR spectra were transferred to MestReNova software (release 6.0.2, Mestrelab Research, Spain) and referenced to *3*-(trimethylsilyl)-propionate-d4 (0 ppm). An integration pattern was defined choosing buckets manually on all the considered spectra in the overlapped form. This procedure permitted to choose buckets as large as to compensate the little chemical shifts fluctuation in each single spectrum and for this reason, it was preferred to the standard automatic bucketing integration that utilizes a bin width of 0.04 ppm. Moreover, in this way, each bucket corresponds to a defined signal or to a group of signals, which simplifies the interpretation of the statistical results. The defined pattern was used for the automatic integration of all the spectra and referred to TSP area (Figure 1). 

### 2.3. Statistical Analysis

Cheese proximate composition and biochemical data were submitted to ANOVA (PROC GLM) adopting a linear model that considered the fixed effects of cows’ feeding system (FS: pasture vs. hay) and ripening time (RT: *Pressato* vs. *Allevo*_4 vs. *Allevo*_6) and their interaction. Pairwise comparisons among levels of the all the factors were performed using Bonferroni correction. The hypotheses of the linear model on the residuals were graphically assessed.

The dataset of the ^1^H NMR normalized integrals formed the matrix that was subjected to targeted multivariate canonical discriminant analysis (CDA) after a preliminary feature selection to avoid redundancy among aqueous variables. The ^1^H NMR features mainly responsible for the prediction of the 6 experimental groups (2 levels of cows’ feeding system per 3 levels of ripening time) were identified using a forward stepwise discriminant analysis (PROC STEPDISC). The further multivariate procedure (PROC CANDISC) was performed on the 13 selected ^1^H NMR features to complete the CDA splitting the total variance in three main canonical functions named CAN 1, CAN 2, and CAN 3. The degree of dissimilarity among the six experimental groups was measured by square Mahalanobis distances (*D*^2^-Mahalanobis). The outcomes of the CDA were plotted to classify the six experimental groups according to the first two canonical functions (CAN 1 and CAN 2) coupled with the loading values of the standardized canonical coefficients of the 13 ^1^H NMR feature predictors. The reliability of the CDA classification model was assessed by a validation on a test set. With the aim to perform the validation (PROC DISCRIMN), the original dataset was split in a training (35 samples) and prediction (20 samples) set by means of a random selection that used as priories probability the proportion of each experimental group of the original dataset. A confusion matrix was built throughout the results of the prediction set and the classification performance was assessed by means of some descriptive statistics such as accuracy, precision, sensitivity, specificity, and Matthews correlation coefficient (MCC) as reported in Bisutti et al., 2019 [16].

All analysis were carried out using the SAS 9.4 software (SAS Institute Inc., Cary, NC, USA) and the scatter plot of the CDA were graphically obtained by using XLStat (Addinsoft, release 2016, New York, USA) and Statistica (TIBCO Software Inc., release 13.4.0.14, Palo Alto, USA) software. The *p*-value threshold for statistical significance was set to 0.05.

## 3. Results and Discussion 

Composition and chemical traits of alpine Asiago PDO did not change according to cows’ feeding system and its interaction with the ripening time, whereas they were instead affected by RT (Table 1). 

The *Pressato* was characterized by the highest moisture content and by the lowest amount of crude protein and fat. As proportion of water-soluble N on the total N, the ripening index can be considered an indicator of the primary proteolysis. This index increased throughout the time of ripening and, according to literature [17,18], it was likely due to the prolonged proteolytic activities of the microbial enzymes on the casein fractions. Regardless of the ripening time fixed effect, the values recorded for the ripening index in the present study were higher than those reported for similar alpine cheeses [18,19]. Behind the role of the chymosin coupled with the milk heating treatment, this difference may be due to the intense proteolytic activity promoted by the selected (*Streptococcus thermophilus* strain) lactic acid bacteria (LAB) used as starter culture. pH was affected (*p* < 0.05) by RT with the highest value observed for *Allevo*_6. Based on the literature [20], the increase of pH in more aged samples could be the consequence of the progressive accumulation of short-chain peptides and free amino acids as well as the lower levels of lactate due to the shortage of lactose, its main fermentative substrate. 

The main purpose of the study was to identify and quantify the water-soluble ^1^H NMR metabolomics profiles of alpine Asiago PDO cheeses obtained from pasture- or hay-based milk at three ripening times and to assess if they differ according to these factors. The ^1^H NMR spectra are dominated by lactic acid signals; others intense signals are those of acetic acid, glycerol, hydrophobic amino acids, ethanol, and citric acid. Signals in the zone 7.0–8.5 ppm are mainly related to aromatic amino acids and others aromatic compounds. Minor signals in the zones 3.3–3.5 and 4.6–5.2 are specific of sugars (Figure 1).

**Figure 2:**
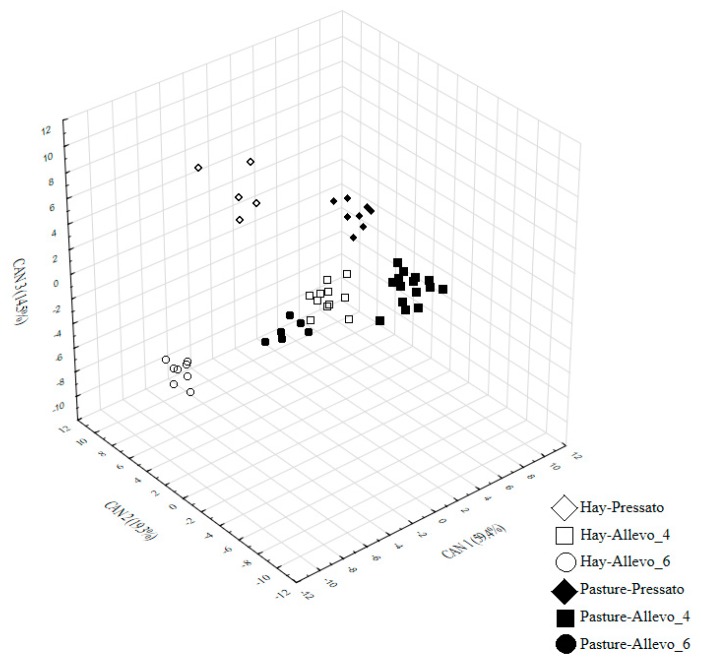
3D scatter plot of the canonical discriminant analysis of the six experimental groups (2 cows’ feeding systems per 3 ripening times) of alpine Asiago PDO samples. *Pressato*: 2-mo of ripening; *Allevo*_4: 4-mo of ripening; *Allevo*_6: 6-mo of ripening.

The supervised multivariate CDA allowed to identify the most informative ^1^H NMR variables for the separation of alpine Asiago PDO samples in a three-dimensional space aiming at maximizing the distances among the a priori defined experimental groups and the independence of the axes (CAN 1 – CAN 3) of the configuration (Figure 2). 

As suggested by the stepwise procedure, the supervised classifier model sorted a restricted pool of 13 informative integrated ^1^H NMR signals that are reported in Table 2. A preliminary ANOVA of the selected 13 informative ^1^H NMR variables is also reported in Table 2. Feeding system significantly (*p* < 0.05) affected the sugar compound A, 2,3-butanediol, lysine, choline and the unknown 1 compound, meanwhile the tyrosine tended (*p* = 0.091) to be different. With regard the RT effect, all the informative ^1^H NMR variables were significantly (*p* < 0.05) influenced. The interaction FS per RT was never significant.

**Figure 3:**
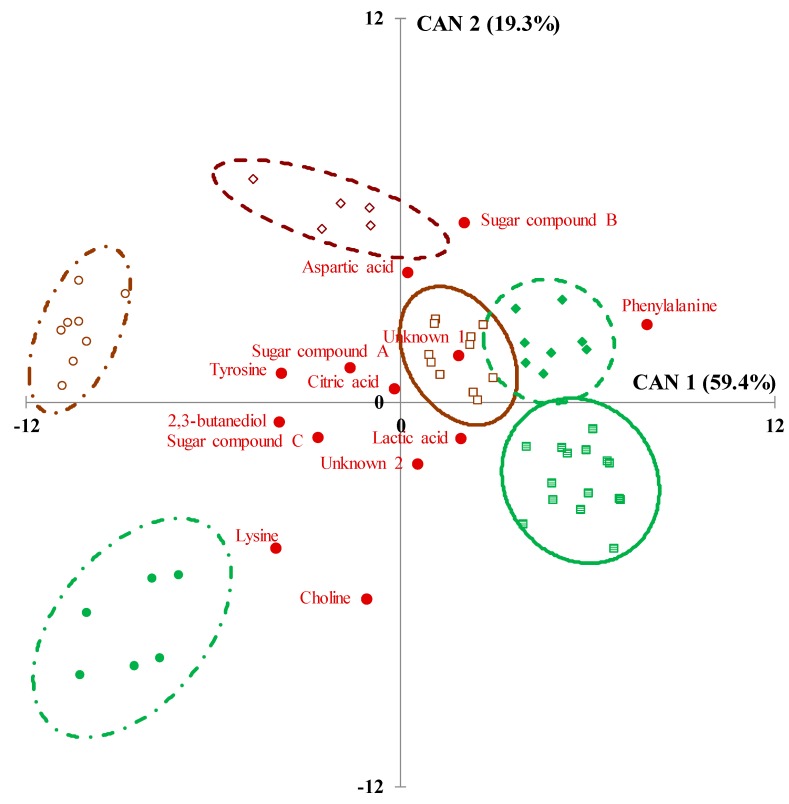
Canonical discriminant analysis (CDA) scatter plot of alpine Asiago PDO samples according to the six experimental groups (2 cows’ feeding systems, FS *per* 3 ripening times, RT). Ninety-five per cent confidence ellipses (0.95-confidence interval) are drawn around each centroid of groupings. The red full circles represent the values of the correlation of the 13 ^1^H NMR signals selected by the preliminary stepwise regression procedure. Green color/full symbols the pasture-based and brown color/empty symbols the hay-based samples. Circles and dotted-point line, *Pressato* (2-mo of ripening); squares and continuous line, *Allevo*_4 (4-mo of ripening); rhombus and dotted line, *Allevo*_6 (6-mo of ripening).

The scatter plot of CDA was coupled with the loading values (by means of the canonical standardized coefficient of the total dataset) of the selected ^1^H NMR variables to identify the discriminative aqueous compounds (Figure 3). The pasture-based samples were characterized by higher level of choline and 2,3 butanediol. Phospholipids are polar lipids with many head-group substituents such as choline, ethanolamine, serine, etc. In fresh forage, the level of phospholipids could reach over 25% (on ether extract basis) with phosphatidyl choline being the main component, even if there is a high forage species and plant organs effect (prevalence in leaves versus stems) [21]. Compared to hay making, the grazing of alpine meadows probably allowed a greater transfer of choline from fresh grass leaves richer in phospholipids.

The 2,3 butanediol represents a metabolite of citrate pathway because of citrate is degraded to pyruvate which then can be converted to 2,3-butanediol via the intermediates α-acetolactate and acetoin. In the study, 2,3 butanediol could be identified as one of the markers of pasture-based *Pressato* cheese, since its amount has shown to disappear with ripening [22]. It is very difficult to find a clear explanation for the significantly higher contents of lysine in pasture-based cheese and tyrosine in the hay-based one. As reported by Mordenti et al. [23], they could arise from the imbalance between amino acid availability and requirement. Moreover, large botanical diversity and environmental eating conditions/forage preservation method (e.g., outdoor/pasture grazing vs. indoor/hay) may influence the milk microbiota originating from teat skin [24] and the following specific proteolytic activities of the microflora conveyed by milk to cheese. Conversely, hay-based samples were more related to the presence of sugar compound A; despite it cannot be unambiguously assigned, good candidates could be galactose because of the signal centered at 3.43 ppm [25]. A noticeable role could be played also by the unknown compound 1, which cannot be also unambiguously assigned (signal centered at 2.93 ppm), even if it is a compound present in a very low content. 

All the ^1^H NMR variables selected by the stepwise procedure of the CDA algorithm were affected by the RT effect (Table 2). The *Pressato* cheese was characterized by the presence of 2,3-butanediol, lysine, tyrosine and sugar compound C (Figure 2). As previously mentioned, 2,3 butanediol is a component of cheese flavor that disappears as the cheese ages [22]. The so-called sugar compound C is a signal centered at 3.96 ppm, a crowded zone mainly related to sugars [25,26]. The signal in this zone mean therefore a residual content of hexose monosaccharides not involved in the main biochemical pathways for lactate or ethanol production yet. The outcomes of the study confirm that phenylalanine is one of the main markers of long-ripened cheese. Moreover, lysine seemed to be related to cheese with a short-medium period of ripening meanwhile tyrosine may be considered a marker of a more prolonged proteolytic process [27]. Proteolytic events occurring during cheese ripening are responsible for the release of free amino acids and peptides and there are affected by a complex process of chemical and biochemical reactions, microbial transformation, water loss, and salt diffusion [17,28]. It is well known that the ripening process leads to a progressive increment of acid organic substances such as lactate and aspartate [29], and the outcomes of the study confirm the preeminent role of these acids as markers of the *Allevo* varieties. The more ripened samples were also negatively correlated with citrate content. In fact, the presence of residual citric acid seemed to be detected in *Pressato* and *Allevo*_4 meanwhile it was at minimal content in *Allevo* samples. Milk citrate is metabolized by LAB into acetate, acetoin and diacetyl, which are aromatic molecular compounds contributing mostly to the flavor of ripened cheese [8]. 

According to the results of the CDA (Wilks’ λ = 0.007, approx. F value = 18.6, df1= 80, df2= 158, *p* < 0.001), a scatter plot of PDO Asiago samples was built according to the standardized canonical coefficients of the first two canonical functions CAN 1 and CAN 2, which explained the 78.7% of total variance (Figure 2). Pasture-based samples of *Pressato* and *Allevo*_4 were grouped on the lower side of the graph, while pasture-*Allevo*_6 samples partially overlapped with hay-*Allevo*_4 ones in the upper-right side. The hay-*Pressato* and hay-*Allevo*_6 samples were spatial arranged in the upper-left side. On the whole, the targeted CDA pointed out a relevant discrimination among experimental groups based on the significant high values of Mahalanobis (D^2^-Mahalanobis) distances that ranged from 45 to 178 (*p* < 0.001). To confirm the discriminative accuracy of the CDA algorithm, a validation was carried out on a prediction set. The outcomes of the validation are reported in the confusion matrix of Table 3 and confirmed that the aqueous ^1^H NMR metabolic profile was a reliable molecular fingerprinting to discriminate pasture- from hay-based Asiago cheese only for the variety *Pressato* with shortest ripening time (Matthews correlation coefficient = 1.00). A progressive loss of accuracy was instead observed in case of more aged *cheese* varieties (Table 3). In fact, within the pasture-based cheese, 29% of *Allevo*_4 samples were misclassified as *Allevo*_6. Moreover, in case of *Allevo*_6, there was a wrong classification according to FS for 25% of pasture-based samples that were assigned to hay-based group. These findings suggest that the metabolic fingerprint of a pasture-based alpine cheese is progressively weakened by the complex set of chemical and biochemical reactions, microbial transformation, water loss and salt diffusion occurring during a prolonged maturation process.

## 4. Conclusions

Proton nuclear magnetic resonance (^1^H NMR) was used to identify and quantify the water-soluble NMR metabolomics profiles of alpine Asiago PDO cheeses obtained from pasture- or hay-based milk at three ripening times. Based on the multivariate CDA, 13 ^1^H NMR water-soluble compounds were selected as the most informative variables for the separation of alpine Asiago PDO samples. Among selected ^1^H NMR variables, choline, 2,3-butanediol, lysine, tyrosine were the most effective aqueous compounds to discriminate cheese samples according to the cows’ feeding system. However, the multivariate CDA revealed that the ^1^H NMR profile was a powerful metabolomic fingerprinting to correctly identify only the samples with the shortest ripening period (*Pressato*). The proportion of correct assignment to the actual feeding system of cheese samples belonging to more aged varieties has shown to decrease due to the chemical and biochemical changes induced by a prolonged maturation process. Therefore, a reliable practical application of this optical-based emerging technology to discriminate alpine pasture Asiago PDO cheeses from hay-based analogues should be restricted to products with a short ripening time. 

## Figures and Tables

**Table 1:** Effect of the ripening time (RT), cows’ feeding system (FS) and their interaction (RT·FS) on proximate composition (g/100 g wet weight), ripening index (RI) and pH of alpine Asiago Protected Designation of Origin (PDO) cheese.

Item	*Pressato*		*Allevo*_4		*Allevo*_6		SEM	*p*-Value		
	Pasture *n* = 6	Hay *n* = 8	Pasture *n* = 15	Hay *n* = 11	Pasture *n* = 9	Hay *n* = 6		RT	FS	RT·FS
Moisture	39.3 ^a^	40.9 ^a^	33.7 ^b^	33.4 ^b^	30.2 ^c^	30.8 ^c^	0.40	<0.001	0.143	0.133
Fat	29.1 ^c^	27.7 ^c^	32.0 ^b^	31.9 ^b^	34.7 ^a^	34.0 ^a^	0.38	<0.001	0.159	0.328
Protein	23.1 ^c^	22.9 ^c^	26.0 ^b^	26.4 ^b^	27.3 ^a^	27.8 ^a^	0.26	<0.001	0.384	0.563
Ash	3.4 ^c^	3.3 ^c^	3.9 ^b^	3.8 ^b^	4.6 ^a^	4.6 ^a^	0.08	<0.001	0.599	0.964
NaCl	0.98 ^c^	0.95 ^c^	1.12 ^b^	1.15 ^b^	1.34 ^a^	1.35 ^a^	0.044	<0.001	0.612	0.845
RI	20.4 ^c^	18.6 ^c^	23.6 ^b^	22.8 ^b^	26.9 ^a^	26.4 ^a^	0.81	<0.001	0.248	0.569
pH	5.52 ^b^	5.54 ^b^	5.54 ^b^	5.56 ^b^	5.61 ^a^	5.64 ^a^	0.019	<0.001	0.241	0.735

*Pressato*: 2-mo of ripening; *Allevo*_4: 4-mo of ripening; *Allevo*_6: 6-mo of ripening. RI: ripening index (water-soluble N over total N × 100). SEM: Standard error of the mean. ^a,b,c^ Means with different superscripts within a row differ (*p* < 0.05).

**Table 2:** Statistical scores of ^1^H NMR predictors of the 6 experimental groups (2 cows’ feeding systems, FS per 3 ripening times, RT) of alpine Asiago PDO according to the stepwise procedure and the ANOVA (main fixed effects FS and RT and their interaction FS per RT).

Step	^1^H NMR Variables	Statistical Parameters of STEPWISE				*p*-Value of ANOVA		
		Wilks’ λ	*F*-Value	*p*-Value	R^2^_partial_	FS	RT	FS·RT
1	Sugar compound A	0.303	21.7	<0.001	0.87	<0.001	<0.001	0.146
2	Sugar compound B	0.282	24.6	<0.001	0.75	0.565	<0.001	0.107
3	2,3-butanediol	0.208	12.6	<0.001	0.57	0.031	<0.001	0.110
4	Sugar compound C	0.182	7.9	<0.001	0.48	0.956	0.001	0.474
5	Lactic acid	0.124	7.7	<0.001	0.46	0.632	0.015	0.354
6	Citric acid	0.110	7.1	<0.001	0.38	0.811	<0.001	0.115
7	Lysine	0.086	4.8	0.002	0.32	0.031	0.001	0.231
8	Unknown 1	0.075	5.8	0.001	0.29	0.021	0.003	0.956
9	Aspartic acid	0.054	4.7	0.002	0.24	0.320	<0.001	0.950
10	Choline	0.033	4.8	0.002	0.20	0.027	0.002	0.747
11	Unknown 2	0.012	4.2	0.008	0.11	0.156	0.008	0.634
12	Phenylalanine	0.008	3.2	0.021	0.12	0.608	0.002	0.974
13	Tyrosine	0.007	4.3	0.005	0.09	0.091	<0.001	0.801

**Table 3:** Descriptive statistics of the validation set based on stepwise feature selection of alpine Asiago PDO ^1^H NMR predictors.

Descriptive Statistics	*Pressato*		*Allevo_4*		*Allevo_6*	
	Pasture *n* = 3	Hay *n* = 2	Pasture *n* = 5	Hay *n* = 2	Pasture *n* = 5	Hay *n* = 3
Accuracy	1.00	1.00	0.89	1.00	0.83	0.94
Precision	1.00	1.00	0.71	1.00	0.75	1.00
Sensitivity	1.00	1.00	1.00	1.00	0.60	0.67
Specificity	1.00	1.00	0.85	1.00	0.92	1.00
MCC	1.00	1.00	0.78	1.00	0.56	0.79

Pressato: 2-mo of ripening; *Allevo*_4: 4-mo of ripening; *Allevo*_6: 6-mo of ripening. MCC: Matthews correlation coefficient. For the significance of the descriptive statistics, see Bisutti et al. 2019 [16].
